# Parasitoid–host eavesdropping reveals temperature coupling of preferences to communication signals without genetic coupling

**DOI:** 10.1098/rspb.2023.0775

**Published:** 2023-08-30

**Authors:** Karina J. Jirik, Jimena A. Dominguez, Iya Abdulkarim, Johanna Glaaser, Emilia S. Stoian, Luis J. Almanza, Norman Lee

**Affiliations:** Department of Biology, St Olaf College, Northfield, MN, USA

**Keywords:** song recognition, temperature coupling, temperature sensitivity, preference function, eavesdropper, walking phonotaxis

## Abstract

Receivers of acoustic communication signals evaluate signal features to identify conspecifics. Changes in the ambient temperature can alter these features, rendering species recognition a challenge. To maintain effective communication, temperature coupling—changes in receiver signal preferences that parallel temperature-induced changes in signal parameters—occurs among genetically coupled signallers and receivers. Whether eavesdroppers of communication signals exhibit temperature coupling is unknown. Here, we investigate if the parasitoid fly *Ormia ochracea*, an eavesdropper of cricket calling songs, exhibits song pulse rate preferences that are temperature coupled. We use a high-speed treadmill system to record walking phonotaxis at three ambient temperatures (21, 25, and 30°C) in response to songs that varied in pulse rates (20 to 90 pulses per second). Total walking distance, peak steering velocity, angular heading, and the phonotaxis performance index varied with song pulse rates and ambient temperature. The peak of phonotaxis performance index preference functions became broader and shifted to higher pulse rate values at higher temperatures. Temperature-related changes in cricket songs between 21 and 30°C did not drastically affect the ability of flies to recognize cricket calling songs. These results confirm that temperature coupling can occur in eavesdroppers that are not genetically coupled with signallers.

## 1. Introduction

The evolution of communication systems can be driven by environmental factors that influence the behaviour of signallers and receivers [1]. Ambient temperature is one such environmental factor that can vary in time and space, with profound effects on locomotion, reproduction, foraging and the communication behaviour of animals [2,3]. This is especially the case for ectotherms where body temperature is regulated by the temperature of the environment. Ambient temperature can affect the production of temporally patterned communication signals [4–7], the transmission of signals through the environment [8], or the functioning of sensory systems [9–11], all of which can impact reproductive success [12,13]. When signals change as a result of changes in ambient temperature, how are the recognition of and preferences for behaviourally salient signals maintained in receivers?

The production of acoustic communication signals involves rhythmic motor activity. Changes in muscle physiology resulting from changes in ambient temperature can affect the temporal [14–24] and spectral [12,21,23,25] features of advertisement signals in a range of animals. In field crickets, males communicate with acoustic signals that are produced by stridulation, a process that involves rubbing together specialized structures on the forewings [26,27]. Contraction of forewing muscles opens and closes the left and right forewings [28,29] with each wing stroke closure generating a sound pulse. As song pulse rate is determined by the rate of muscle contractions, an increase in ambient temperature can lead to an increase in song pulse rates [15,16,30].

Temperature-dependent changes in acoustic communication signals can potentially interfere with the ability of receivers to recognize conspecifics and evaluate signals to make appropriate behavioural decisions (e.g. mate choice) [12,17,28]. As a solution to this problem, receivers may exhibit changes in signal preferences that parallel temperature-dependent changes in signal features [17,20,24,25,29]. This temperature coupling ensures that signal recognition is maintained, and that receivers can remain selective for high quality mates, which will allow for sexual selection to operate across a range of ambient temperatures. Accordingly, temperature coupling may be an adaptive function to ensure that thermal changes in signal production are matched with receiver signal recognition and preferences.

In the field cricket *Gryllus firmus*, the chirp and pulse rate of calling songs vary with temperature, and females exhibit a parallel change in preferences [20]. One proposed explanation for temperature coupling is that signallers and receivers may share common neural elements due to genetic coupling [28]. Through a shared pleiotropic locus, or tight linkage between a sexual trait (song pulse rate) and preference loci, ambient temperature would simultaneously affect signal features and preferences for such features [31,32]. While this might be a plausible explanation for intended receivers that are genetically linked to the signaller, whether and how unintended receivers might exhibit signal preferences that parallel temperature-dependent changes in song features remains to be examined.

The parasitoid fly *Ormia ochracea,* an unintended receiver of cricket calling songs, relies on directional hearing to eavesdrop on the calling songs of field crickets in search of appropriate host species [33–35]. Song recognition is based on evaluating the temporal patterning of sound pulses [35,36]. In Florida, *Gryllus rubens* is the preferred cricket host [37,38], which produces calling songs characterized by a pulse rate of about 45–50 pulses per second (pps), but varies with ambient temperature [39]. Floridian *O. ochracea* exhibit pulse rate preference functions that peak at the same range of pulse rates [36]. Upon detection of appropriate calling songs, gravid female flies will perform flying and walking phonotaxis to the source location [40–42], where they deposit first instar planidia near or on top of the host. The planidia will then burrow into the cricket, develop for a period of 7 to 10 days, emerge from the cricket, pupate, and finally hatch as an adult fly [43–45]. Reproduction in *O. ochracea* crucially depends on the ability of flies to recognize and localize host cricket calling songs.

In this study, we investigate whether temperature coupling can arise between two species that are disparately related and not genetically coupled. Specifically, we study whether temperature-dependent changes in song pulse rates affect the ability of *O. ochracea* to recognize and localize appropriate host cricket calling songs. While *O. ochracea* are not genetically coupled to crickets, gravid female flies are expected to evolve signal preferences that parallel thermal-induced changes in signal features. We specifically test the hypothesis that preferences for signal features exhibited by eavesdroppers are coupled to temperature-dependent changes in signal parameters. According to this hypothesis, we expect that as the pulse rate of calling songs increases with ambient temperature, the peak in pulse rate preference functions exhibited by Floridian *O. ochracea* will show a concomitant shift to higher pulse rate values. To address this hypothesis, we measured walking phonotactic responses of *O. ochracea* when subjected to different ambient temperatures in response to calling songs that varied in pulse rates. Our results indicate that the peak of pulse rate preference functions in Floridian *O. ochracea* shift with ambient temperature. Pulse rate preference functions also appear to broaden at higher temperatures, indicating lower levels of pulse rate selectivity at higher temperatures.

## 2. Material and methods

### (a) Animals

Behavioural walking phonotaxis experiments were conducted on gravid female *O. ochrace*a from a laboratory colony that was originally derived from Gainesville, Florida. Flies were reared in a temperature-, humidity- and light-controlled environmental chamber (Power Scientific, Inc., model DROS52503, Pipersville, PA) set to a 12 h : 12 h light/dark cycle, 25°C, at 75% humidity, and provided butterfly nectar solution (The Birding Company, MA) ad libitum.

### (b) Experimental setup

Walking phonotactic responses were recorded from flies tethered and held on top of a spherical treadmill system previously described in [46] and [36]. This treadmill system consists of a spherical ping-pong ball held afloat in a custom holder with a continuous air stream. Rotations of the spherical treadmill actuate a modified optical mouse sensor (ADNS 2620, Avago Technologies, USA) that is situated directly below the treadmill. This optical mouse sensor registers motion as changes in *x* and *y* pixel units at a sampling rate of 2160 Hz [46]. In a previous study [36], we confirmed that *O. ochracea* never causes the spherical treadmill to rotate on its axis and thus a single sensor system is sufficient for recording *O. ochracea* walking phonotactic responses. Data captured from the treadmill system were synchronized with sound presentation using software (StimProg V6) developed in MATLAB (R2018a, The MathWorks Inc., USA) that interfaced with National Instruments data acquisition hardware (NI USB-6363, USA). Changes in *x* and *y* pixel values were calibrated to real-world distances in centimetres by measuring the actual displacement of the spherical treadmill in footage captured using a high-speed camera (Chronos 1.3 High-speed Camera, 1000 frames per second, Krontech, Canada). Images from this high-speed camera were imported into ImageJ (v. 1.51), and the displacements of markers on the spherical treadmill were measured relative to a known distance. This treadmill system was situated 25 cm away from two silk-dome tweeters (1–1/8 Dayton Audio Classic Series DC28FS-8, USA), which were located at ±45° azimuth (to the left and right of the treadmill system) relative to the forward (0° azimuth) direction of a tethered fly. This experimental setup was situated on a vibration isolation table (TMC) and located in an acoustically dampened sound chamber (Wenger Soundlok, USA). Ambient temperature in the sound chamber was controlled by the building heating, ventilation and air conditioning (HVAC) system and fine-tuned by using a Lasko space heater (model CT2272, China). Ambient temperature near the animal was verified with a thermometer suspended 10 cm directly above the treadmill system. This approach allowed temperature control to a precision of ±1°C.

### (c) Acoustic stimuli

Acoustic stimuli consisted of synthetically produced cricket songs that were created in MATLAB. Following our previous work on tethered walking phonotaxis [35,36], cricket songs were composed of tonal sound pulses with a carrier frequency of 5 kHz and a temporal envelope that was shaped with 1 ms linear onset and offset ramps. The 1 ms onset and offset ramps were designed to avoid abrupt temporal envelope changes that may introduce frequency distortions in the signal playback. Song pulse rate was varied by changing pulse durations and interpulse intervals in equal amounts to maintain a 50% duty cycle such that the acoustic energy would be equal across pulse rate songs for a given stimulus duration. The total stimulus duration for different pulse rate songs was maintained at a 1-s duration to ensure equal acoustic energy across all test stimuli. In total, eight different pulse rate songs were used as test stimuli (20 to 90 pps, in increments of 10 pps). These digital signals were converted to analogue signals using the digital-to-analogue converter of the National Instruments data acquisition device, amplified using an audio amplifier (Crown XLS1002 Drive Core 2, USA), attenuated with programmable attenuators (Tucker Davis Technologies System 3 PA5, USA), and broadcast through the speakers. Speakers were calibrated to 75 dB SPL (rel. 20 µPa, fast, Z-weighting) at the location of the fly's head using a probe microphone (Hottinger Brüel & Kjær, Type 4182, Denmark) connected to a sound level meter (Hottinger Brüel & Kjær, Type 2250, Denmark).

### (d) General experimental protocol

Prior to behavioural experiments, flies were anaesthetized on ice for 5 min and subsequently attached to a tether using a custom low-melting point wax (combination of bee's wax, rosin and Sally Hansen wax kit). After this mounting procedure, flies were allowed to acclimate to the testing environment for a period of 15–30 min before behavioural testing. Walking phonotaxis experiments were conducted in the dark under IR illumination so that walking responses could be monitored via the digital display on the rear of the high-speed camera equipped with a macro lens (Nikon AF MICRO NIKKOR 105 mm f/2.8 D, Japan).

Song pulse rate preferences at different ambient temperatures were tested within subjects. Testing for each subject started at a temperature (21, 25 or 30°C) that was randomly determined. A 50 pps 1 s control song was presented from the left speaker, followed by the presentation of the same control song from the right speaker. Test songs that varied in pulse rates were initially presented from one speaker for the first 0.5 s and then switched and presented from the other speaker for the remaining 0.5 s of stimulus broadcast. Whether the left or right speaker was leading in the stimulus broadcast was chosen at random. Each pulse rate test stimulus was tested three times, resulting in a total of 24 test stimuli presentations at each temperature condition. The order of test stimuli presentations was fully randomized. A particular temperature treatment condition ended with a final round of testing the control stimulus from the left speaker, followed by the right speaker. This testing procedure was replicated at the other two ambient temperatures.

Walking phonotactic responses were considered to be valid if total walking distance exceeded 1 cm. For every three consecutive non-responses to test stimuli, we tested a control stimulus to ensure that the subject was still motivated to respond. If the subject did not respond to this control stimulus, the experiment ended. These data were considered to be incomplete and were excluded from the data analysis. To control for carry-over effects of hearing a previous stimulus, subjects were given 1 min of rest before testing the next stimulus.

### (e) Data analysis

Response latencies were determined as the time between stimulus presentation and the first indication of movement (any changes in *x* or *y* pixel values). Steering and forward velocities were determined as changes in *x* and *y* pixel values over time, respectively. We calculated cumulative total walking distance as:

$$\mathrm{distance}=\sqrt{{\left(x_{2}-x_{1}\right)}^{2}+{\left(y_{2}-y_{2}\right)}^{2}},$$

summed across sample points collected during the 1.5 s of data capture. The instantaneous angular heading (in degrees) was determined by converting Cartesian *x* and *y* values to polar coordinates by computing the inverse tangent of *y* divided by *x* (angular heading = arctan(*y*/*x*)). Angular heading was quantified in response to speakers located at ± 45° azimuth (−45°: left speaker, +45°: right speaker). We calculated the mid-response angular heading at the mid-point of stimulus broadcast (0.5 s into data capture) which occurred when the broadcast location was switched from the initial location to the subsequent location (e.g. left to right, or right to left). Responses to stimuli where the initial broadcast occurred from the left speaker first were mirrored and reflected across 0° (forward direction). Total walking distance, peak steering velocity and angular heading were incorporated into calculating the phonotaxis performance index described in [36]. Index values range from 0 to >1. A phonotaxis performance index of 0 indicates poor performance, 1 indicates performance equivalent to responses to the standard song, >1 indicates performance ‘better’ than responses to the control song (e.g. higher steering velocity, longer distance).

We used separate two-way repeated measures ANOVAs to examine the effects of song pulse rate and ambient temperature on (1) response latency, (2) steering velocity, (3) total walking distance, and (4) the phonotaxis performance index. Greenhouse–Geisser corrections were applied where assumptions of sphericity were violated. The *p*-values from multiple *post hoc* pairwise comparisons were corrected with Bonferroni adjustments. All statistical analyses were conducted in SPSS Statistics (ver. 19, IBM Corporation, USA).

As PFunc developed by Kilmer *et al*. [47] did not allow us to generate appropriately fitted curves to capture preference functions in this current study, we created a MATLAB script that generated preference functions based on fitting cubic splines to preferences values. This analysis allowed us to visualize the peak preference and tolerance. Tolerance values describe the broadness or width of preference functions and were calculated as the width of the preference function at 25% below the peak value.

## 3. Results

To determine how cricket song pulse rate preferences vary with ambient temperature, we recorded tethered walking phonotaxis from 18 Floridian *O. ochracea* using a high-speed spherical treadmill system [46]. Flies were subjected to synthetic cricket songs that varied in pulse rates (20 to 90 pps, in increments of 10 pps), under three different ambient temperatures (21, 25 and 30°C).

When flies engaged in walking phonotaxis, they responded with mean latencies that ranged from 16.51 ms to 172.53 ms at 21°C, 15.97 ms to 290.00 ms at 25°C and 14.58 ms to 158.33 ms at 30°C. Response latencies did not vary significantly with pulse rates (rmANOVA: *F*_7,119_ = 1.685, *p* = 0.119, $\eta_{p}^{2}=0.09$), ambient temperature (*F*_2,34_ = 0.792, *p* = 0.461, $\eta_{p}^{2}=0.044$), nor was there a significant interaction between pulse rate and ambient temperature (*F*_14,238_ = 0.953, *p* = 0.502, $\eta_{p}^{2}=0.053$).

**Figure 1.**
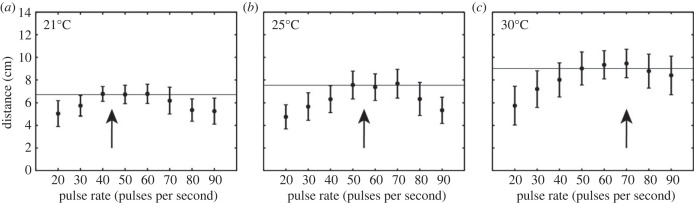
Total walking distance varied with song pulse rate and ambient temperature. Total walking distance (cm) (mean ± SEM) as a function of song pulse rate (pps) at (*a*) 21°C, (*b*) 25°C, (*c*) 30°C. Arrows denote expected cricket song pulse rate at the specified ambient temperature (21°C: 44.28 pps, 25°C: 55.53 pps, 30°C: 69.34 pps) based on [39]. Horizontal lines indicate total walking distance at 50 pps, which represents the pulse rate of the standard song in the current study.

We found a significant main effect of pulse rate (rmANOVA: *F*_7,119_ = 11.167, *p* < 0.001, $\eta_{p}^{2}=0.396$), temperature (*F*_2,34_ = 20.275, *p* < 0.001, $\eta_{p}^{2}=0.544$), and the interaction between pulse rate and temperature on total walking distance (*F*_14,238_ = 2.946, *p* < 0.001, $\eta_{p}^{2}=0.148$). Similar to previously published work [36], flies walked significantly more for a range of pulse rates between 40 and 70 pps compared to higher or lower pulse rates (figure 1, table 1). Flies also had a tendency to walk significantly greater distances at 30°C than compared to 21°C (*F*_1,17_ = 27.822, *p* < 0.001, $\eta_{p}^{2}=0.621$), but walking distances did not significantly differ at 21°C compared to 25°C (*F*_1,17_ = 2.249, *p* = 0.152, $\eta_{p}^{2}=0.117$). A difference contrast (reverse helmert) revealed that flies in an ambient temperature of 30°C responded with greater walking distances at higher pulse rates than compared to responses at 21°C (electronic supplementary material, table S1). This temperature by pulse rate interaction was less pronounced when comparing responses at 21°C versus those at 25°C. At 21°C, walking distances peaked between 40 and 60 pps, reaching a maximum of 6.77 ± 0.44 cm (mean ± SEM) in response to a 60 pps song. At 25°C (figure 1*b*), total walking distances across most pulse rates were slightly elevated compared to those observed at 21°C (figure 1*a*). Walking distance peaked between 50 and 70 pps, with a peak distance of 7.67 ± 0.68 cm in response to 70 pps (figure 1*b*). At 30°C (figure 1*c*), highest walking distances occurred for a range of pulse rates from 50 to 70 pps, with a maximum of 9.45 ± 0.66 cm (mean ± SEM) in response to the 70 pps song.

**Table 1.** Results from analyses of variance of phonotaxis response metrics in response to different pulse rate songs at different ambient temperatures.

response metric	effect	d.f.	*F*	*p*	partial *η*^2^
latency	pulse rate	7,119	1.685	0.119	0.090
	temperature	2,34	0.792	0.461	0.044
	pulse rate × temperature	14,238	0.953	0.502	0.053
distance	pulse rate	7,119	11.167	<0.001	0.396
	temperature	2,34	20.275	<0.001	0.544
	pulse rate × temperature	14,238	2.946	<0.001	0.148
forward velocity	pulse rate	7,119	7.018	<0.001	0.292
	temperature	2,34	21.736	<0.001	0.561
	pulse rate × temperature	14,238	2.238	0.007	0.116
steering velocity	pulse rate	7,119	10.224	<0.001	0.362
	temperature	2,34	11.46	<0.001	0.389
	pulse rate × temperature	14,238	3.331	<0.001	0.156
phonotaxis index	pulse rate	7,119	8.695	<0.001	0.338
	temperature	2,34	3.318	0.048	0.163
	pulse rate × temperature	14,238	2.247	0.07	0.117

**Figure 2.**
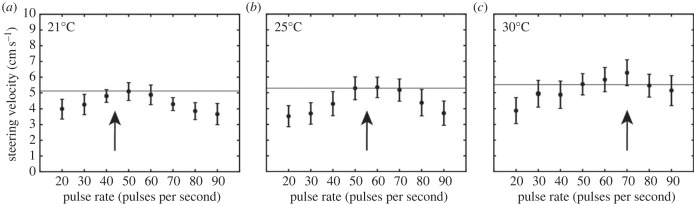
Peak steering velocity varied with song pulse rate and ambient temperature. Peak steering velocity (cm s^−1^) (mean ± SEM) as a function of song pulse rate (pps) at (*a*) 21°C, (*b*) 25°C, (*c*) 30°C. Arrows denote expected cricket song pulse rate at the specified ambient temperature (21°C: 44.28 pps, 25°C: 55.53 pps, 30°C: 69.34 pps) based on [39]. Horizontal lines indicate peak steering velocity at 50 pps, which represents the pulse rate of the standard song in the current study.

We also found a significant main effect of pulse rate (rmANOVA: *F*_1,119_ = 10.224, *p* < 0.001 $\eta_{p}^{2}=0.362$), temperature (*F*_1,34_ = 11.46, *p* < 0.001, $\eta_{p}^{2}=0.362$), and the interaction between pulse rate and temperature on average peak steering velocity (*F*_14,234_ = 3.331, *p* < 0.001, $\eta_{p}^{2}=0.156$). In general, flies exhibited significantly increased average peak steering velocities for a range of pulse rates between 50 and 70 pps compared to higher or lower pulse rates (figure 2, table 1). Flies exhibited greater peak steering velocities at 30°C than compared to 21°C (*F*_1,17_ = 13.263, *p* = 0.002, $\eta_{p}^{2}=0.438$), but did not differ for 21°C compared to 25°C (*F*_1,17_ = 0.034, *p* = 0.855, $\eta_{p}^{2}=0.002$). Analysis of difference contrasts revealed that flies responded with greater peak steering velocities in response to 60 and 50 pps songs relative to all other pulse rate songs at 25°C compared to responses at 21°C (electronic supplementary material, table S1). When steering responses at 30°C were compared to those at 21°C, elevated steering responses occurred only at the higher pulse rates of 70 and 80 pps (electronic supplementary material, table S1). At 21°C, steering velocity peaked between 40 and 60 pps, reaching a maximum at 50 pps song (5.18 ± 0.29 cm s^−1^). At 25°C, steering velocity was greatest from 50 to 70 pps, and reached a peak at 60 pps song (5.44 ± 0.33 cm s^−1^). At 30°C, peak steering velocity was greatest from 60 to 80 pps, with a peak at the 70 pps song (6.46 ± 0.45 cm s^−1^).

Mean angular heading varied with song pulse rate preferences (electronic supplementary material, figure S1). There was a greater tendency for flies to perform walking phonotaxis with angular headings that approached 45° (location of the speaker) at the most preferred pulse rates (40–70 pps). However, angular headings were highly variable in response to most pulse rates and ambient temperature levels, suggesting that the accuracy in localizing the speaker location may not depend on temperature.

**Figure 3.**
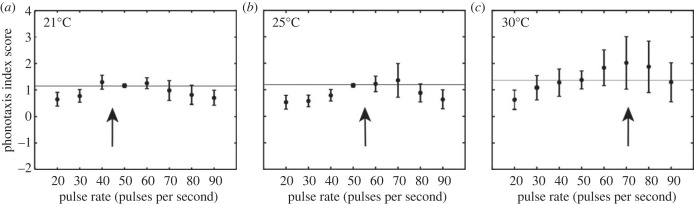
Phonotaxis performance index score varied with song pulse rate and ambient temperature. Phonotaxis performance index scores (mean ± SEM) as a function of song pulse rate (pps) at (*a*) 21°C, (*b*) 25°C, (*c*) 30°C. Arrows denote expected cricket song pulse rate at the specified ambient temperature (21°C: 44.28 pps, 25°C: 55.53 pps, 30°C: 69.34 pps) based on [39]. Horizontal lines indicate phonotaxis performance index score at 50 pps, which represents the pulse rate of the standard song in the current study.

We examined the effects of temperature on pulse rate preferences and overall phonotactic performance using our previously described phonotaxis performance index [36]. There was a significant main effect of pulse rate (*F*_7,119_ = 8.695, *p* < 0.001, $\eta_{p}^{2}=0.338$) and temperature (*F*_2,34_ = 3.318, *p* = 0.048, $\eta_{p}^{2}=0.163$), while the interaction between pulse rate and temperature on the phonotaxis performance index approached significance (*F*_14,238_ = 2.247, *p* < 0.07, $\eta_{p}^{2}=0.117$). Flies had significantly higher phonotaxis performance index scores for a range of pulse rates between 40 and 80 pps compared to those at higher or lower pulse rates (figure 3, table 1). Analysis of difference contrasts revealed that when 25°C was compared to 21°C, flies responded with higher phonotactic performance at most intermediate pulse rate values (e.g. 70, 50, 40 pps; see electronic supplementary material, table S1). At 30°C compared to 21°C, peak performance was highest in response to 70 pps relative to all lower pulse rates (electronic supplementary material, table S1).

**Figure 4.**
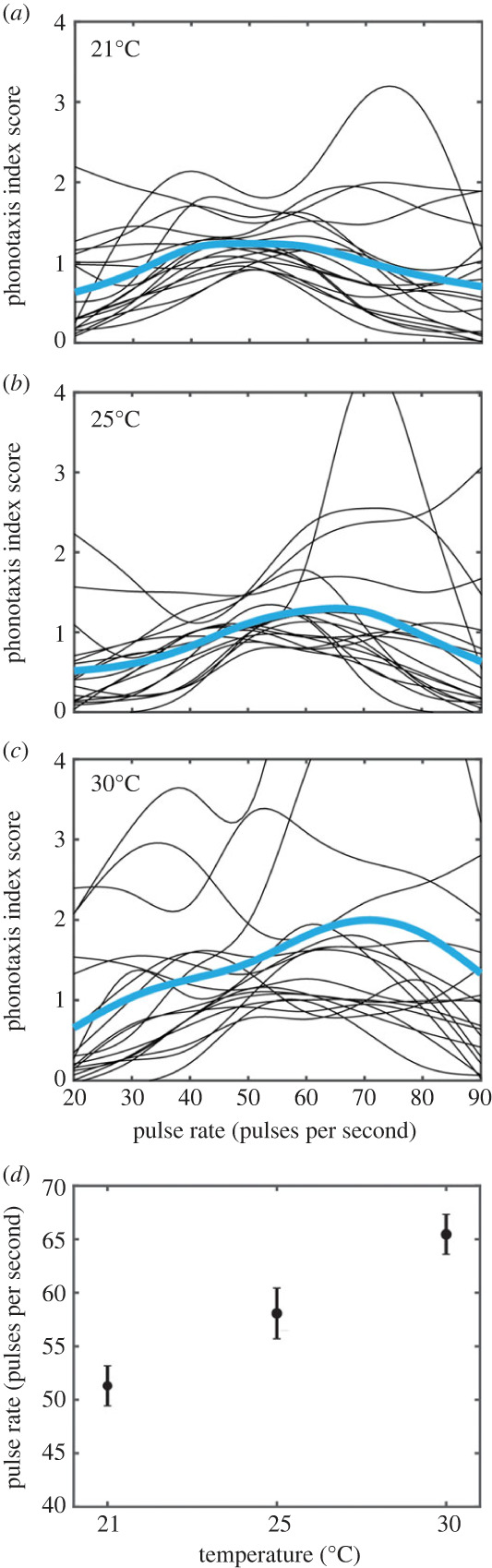
Pulse rate preference functions based on phonotaxis performance index scores as a function of ambient temperature. Preference functions were developed as cubic splines fitted to phonotaxis performance index scores as they varied with song pulse rates at (*a*) 21°C, (*b*) 25°C, (*c*) 30°C. Black lines represent individual preference functions (*n* = 18 flies) while blue lines represent mean pulse rate preference function. Axes in (*a*–*c*) scaled to emphasize mean preference functions, which resulted in the cropping of some individual preference curves. Peak pulse rate preference values were extracted from individual curves and the mean ± SEM peak pulse rate preference is plotted as a function of ambient temperature in (*d*).

To characterize signal preference functions that relate to finding suitable host crickets, we developed a custom MATLAB script with similar functionality to PFunc [47]. This MATLAB script allowed us to fit cubic splines to phonotaxis performance index scores as a function of song pulse rates (figure 4). The fitted preference functions across individuals varied in shape. For example, some flies had preference functions that were linear with peak phonotaxis performance at extreme pulse rate values (e.g. 20 or 90 pps). Some individuals had bimodal preference functions, and others had preference functions that had peaks at intermediate values. Of the 18 flies, 61% (11/18) demonstrated a shift in the peak pulse rate preference to higher pulse rate songs at higher ambient temperatures, and 39% (7/18) of flies demonstrated an increase in tolerance as seen by an increase in the broadness of their pulse rate preference functions.

Among 72% of the flies tested (13/18), peaks were successfully identified from fitted preference functions across pulse rates and all ambient temperature conditions. There was a significant main effect of temperature on the peak in pulse rate preference (rmANOVA: *F*_2,34_ = 13.12, *p* < 0.0001, $\eta_{p}^{2}=0.522$). At an ambient temperature of 21°C, the pulse rate preference function had a peak at 51.31 ± 1.87 pps (mean ± SEM) (figure 4*a*,*d*). At 25°C, the peak preference value increased to a significantly higher pulse rate of 58.08 ± 2.36 pps (figure 4*b*,*d*, *F*_1,12_ = 5.047, *p* = 0.044, $\eta_{p}^{2}=0.296$). When the ambient temperature was increased to 30°C, the peak of the pulse rate preference function increased to 65.46 ± 1.87 pps, which was significantly different from the peak preference exhibited at 21°C (figure 4*a*,*d*, *F*_1,12_ = 31.97, *p* < 0.001, $\eta_{p}^{2}=0.727$).

Shifts in the phonotaxis performance index peak pulse rate preference as a function of ambient temperature also occurred along with a non-significant, but a trending tendency in the broadening of preference functions (*F*_2,28_ = 3.008, *p* = 0.066, $\eta_{p}^{2}=0.177$). At 21°C, tolerance values were 27.86 ± 1.49 (mean ± SEM). These tolerance values increased to 33.92 ± 3.81 and 39.24 ± 3.81 when the ambient temperature was increased to 25°C and 30°C, respectively.

## 4. Discussion

As temporal features of cricket calling songs vary with ambient temperature [15,16,20,39], we sought to determine if temperature-dependent changes in song patterns would affect the ability of *O. ochracea* to recognize or alter preferences for the calling songs of suitable host cricket species. This was assessed by examining the effects of ambient temperature on several key walking phonotactic response metrics. Consistent with our previous work [36], with the exception of response latencies, all other metrics (total distance, peak steering velocity, angular heading, phonotaxis performance index) varied with song pulse rate. At 21°C, the magnitude of these response metrics tended to peak at around 50–70 pps, and declined at higher and lower pulse rates. At the highest ambient temperature tested (30°C), these response metrics generally increased and reached a plateau in response to higher pulse rates, resulting in preference functions with a ‘high-pass' appearance. Over this same range of ambient temperatures, the calling song pulse rate of *G. rubens* has been demonstrated to increase from 40 to 70 pps [39]. Our results revealed a change in the peak of pulse rate preference functions that supports ‘temperature coupling’ as seen in intraspecific acoustic communication systems among some orthopterans and anurans [14,17,19,20,28]. As *O. ochracea* and *G. rubens* are disparately related and thus are not genetically coupled, we can rule out pleiotropy as a cause for temperature coupling.

Walker [37] performed a series of sound trap experiments in the field to determine if the ‘acoustic template’ of *O. ochracea* varied with ambient temperature. In these experiments, a *G. rubens* calling song with a temperature-dependent pulse rate that was consistent with the ambient temperature was broadcast from one sound trap, while a calling song with a pulse rate that was temperature adjusted to be either 4°C above or below (equivalent to 11 pps higher or lower than the temperature-appropriate song pulse rate) the ambient temperature was broadcast from the alternative sound trap. Under these experimental conditions, the sound trap broadcasting a pulse rate song consistent with the ambient temperature attracted the most flies, which suggested that the internal acoustic template for song recognition in *O. ochracea* is temperature-dependent. Our current results extend these past findings in several important ways. First, we applied a robust and highly sensitive phonotactic measurement approach (tethered walking phonotaxis on a high-speed treadmill system) along with a repeated measures design to dynamically characterize pulse rate selectivity for a broader range of pulse rates (20–90 pps). This allowed for the use of a function-valued approach [47] to characterize the preference functions describing pulse rate selectivity. Second, temperature-related changes in these preference functions demonstrate a behavioural mechanism that suggest that hearing in *O. ochracea* is temperature sensitive*.* These signal preference functions across different ambient temperatures are consistent with perceptual neurosensory mechanisms involved in song recognition that are expected to vary with ambient temperature. Finally, while most studies have focused on the effects of ambient temperature on signal production [5,15,16,23,39], our current work represents the first to quantify signal perception (pulse rate selectivity) and how it varies with ambient temperature in an unintended receiver.

An increase in ambient temperature caused a shift in the peak of the pulse rate preference functions, along with a slight tendency for an increase in tolerance for a wider range of pulse rates. Consistent with previous findings [36], our current results demonstrate that *O. ochracea* prefers cricket songs with a pulse rate of 50 pps at 21°C. At higher temperatures, *O. ochracea* walked faster and farther, which contributed to higher phonotaxis performance index values in response to generally less preferred higher pulse rate songs (>70 pps). Such increases in these walking response metrics indicate an increase in the overall phonotactic responsiveness and the broadening of acceptable pulse rates that can elicit phonotactic behaviour. Together, these temperature-dependent changes resulted in preference functions that were ‘closed-ended’ at 21°C, but more ‘open-ended’ at 30°C.

Several gaps in our understanding of song pattern recognition and signal preference in *O. ochracea* still remain. It is known that *O. ochracea* occurs in parts of Mexico, in the southern USA, and on some Hawaiian Islands [48]. In each of these regions, *O. ochracea* are behaviourally specialized to prefer specific host cricket species [38], and it is clear that some populations of *O. ochracea* may recognize and use multiple host cricket species [37,49,50]. Species-specific differences in cricket songs are largely based on the temporal patterning of sound pulses [48]. How fine-scale temporal features contribute to song preferences and song recognition is unknown. For example, Floridian *O. ochracea* prefer cricket songs within a range of pulse rates (40–50 pps) [35,36]. This pulse rate preference may be based on a preference for a particular pulse duration, interval between pulses or different combinations of durations and intervals that make up a particular pulse period [51]. Whether different populations of *O. ochracea* evaluate the same temporal features (e.g. durations, intervals, pulse periods) but prefer a different range of values, and how temperature may affect song recognition across populations, remain to be determined. In the laboratory, we have now demonstrated that the ‘acoustic template’ of Floridian *O. ochracea* may be broad enough to allow for host song recognition across a range of temperatures, but future work should address to what extent *O. ochracea* encounter this range of temperatures during their phonotactic approach in nature.

Temperature coupling between signallers and intended receivers may depend on common genetics and neural architecture [28] among conspecifics to adaptively maintain signal recognition between conspecifics across a wide range of ambient temperatures. However, in some instances, temperature coupling seems to be an emergent property of cellular processes that change with temperature in ways that may not contribute to signal recognition [52]. For example, in the short-horned grasshopper *Omocestus viridulus*, increases in ambient temperature causes chirp duration preferences to diverge from actual song durations produced at higher temperatures [53]. In the acoustic moth *Achroia grisella*, the pulse-pair rate produced by males increases with temperature (18–30°C tested), and female pulse-pair rate response threshold also increases with temperature, but the male pulse-pair rates were all above the recognition threshold at all temperatures tested [52]. By contrast, gravid female *O. ochracea* are not genetically coupled with their host crickets. Any temperature-dependent changes in song recognition and signal preferences may be based on common temperature effects on underlying cellular processes, which may include auditory neurons that are involved in song pattern recognition [54]. Such mechanisms underlying the receiver's psychology (including unintended receivers), would be under strong selection to either be tuned, or have sufficient broadness. As reproductive success in *O. ochracea* is highly dependent on recognizing and localizing suitable host cricket calling songs, *O. ochracea's* acoustic template for host cricket song recognition should be broad and flexible to account for small changes in temporal features across different ambient temperatures.

Eavesdropping predators, parasites and parasitoids are widespread and commonly rely on acoustic communication signals to locate their prey or host [55,56]. These signals can be characterized by spectrotemporal fluctuations that occur over time and can vary with ambient temperature. Under temperature-varying conditions, sensory mechanisms in eavesdroppers that are involved in signal recognition will need to compensate for temperature-related changes in signal features to maintain appropriate behavioural decisions. This is especially the case for eavesdropping specialists with sensory specializations that have coevolved to exploit signal features of specific host species and where mis-recognition is reproductively costly [55]. Our work has revealed that temperature coupling between a host signal feature (e.g. song pulse rate) and preferences exhibited by an eavesdropping specialist (*O. ochracea*) can occur even in the absence of genetic coupling and pleiotropy. We expect other acoustically orienting parasitoids that rely on the recognition of temporally patterned signals to also exhibit temperature coupling. Within the tribe Ormiini (Family Tachinidae), this may include some 70 parasitoid species across three genera (*Ormia*, *Therobia*, and *Homotrixa*) that eavesdrop on crickets and katydids. Within the tribe Emblemasomatini (Family Sarcophagidae), this may include 17 species that parasitize cicada [57]. At present, the precise mechanism by which temperature coupling is achieved and how widespread this may occur in eavesdroppers remains unknown and require further investigation.

## Data Availability

Data can be accessed through the Dryad Digital Repository: https://doi.org/10.5061/dryad.jwstqjqfs [58]. The data are provided in electronic supplementary material [59].

## References

[1] Endler JA. 1992 Signals, signal conditions, and the direction of evolution. Am. Nat. **139**, S125-S153. (10.1086/285308)

[2] Abram PK, Boivin G, Moiroux J, Brodeur J. 2017 Behavioural effects of temperature on ectothermic animals: unifying thermal physiology and behavioural plasticity. Biol. Rev. **92**, 1859-1876. (10.1111/brv.12312) 28980433

[3] Leith NT, Macchiano A, Moore MP, Kasey D Fowler-Finn KD. 2021 Temperature impacts all behavioral interactions during insect and arachnid reproduction. Curr. Opin. Insect Sci. **45**, 106-114. (10.1016/j.cois.2021.03.005) 33831604

[4] Edmunds Jr LN. 1963 The relation between temperature and flashing intervals in adult male fireflies, *Photinus pyralis*. Ann. Entomol. Soc. Am. **56**, 716-718. (10.1093/aesa/56.5.716)

[5] Shimizu I, Barth FG. 1996 The effect of temperature on the temporal structure of the vibratory courtship signals of a spider (*Cupiennius salei* Keys.). J. Comp. Physiol. A **179**, 363-370. (10.1007/BF00194990)

[6] Dunlap KD, Smith GT, Yekta A. 2000 Temperature dependence of electrocommunication signals and their underlying neural rhythms in the weakly electric fish, *Apteronotus leptorhynchus*. Brain. Behav. Evol. **55**, 152-162. 10899709 10.1159/000006649

[7] Rosenthal MF, Elias DO. 2019 Nonlinear changes in selection on a mating display across a continuous thermal gradient. Proc. R. Soc. B **286**, 20191450. (10.1098/rspb.2019.1450) PMC6661355 31337317

[8] Iglesias-Carrasco M, Head ML, Martín J, Cabido C. 2018 Increased temperature disrupts chemical communication in some species but not others: the importance of local adaptation and distribution. Ecol. Evol. **8**, 1031-1042. (10.1002/ece3.3646) 29375776 PMC5773306

[9] Linn CE, Campbell MG, Roelofs WL. 1988 Temperature modulation of behavioural thresholds controlling male moth sex pheromone response specificity. Physiol. Entomol. **13**, 59-67. (10.1111/j.1365-3032.1988.tb00909.x)

[10] Stiebler IB, Narins PM. 1990 Temperature-dependence of auditory nerve response properties in the frog. Hear. Res. **46**, 63-81. (10.1016/0378-5955(90)90140-K) 2380128

[11] Franz A, Ronacher B. 2002 Temperature dependence of temporal resolution in an insect nervous system. J. Comp. Physiol. A **188**, 261-271. (10.1007/s00359-002-0298-6) 12012097

[12] Brandt EE, Kelley JP, Elias DO. 2018 Temperature alters multimodal signaling and mating success in an ectotherm. Behav. Ecol. Sociobiol. **72**, 191. (10.1007/s00265-018-2620-5)

[13] Brandt EE, Rosenthal MF, Elias DO. 2020 Complex interactions between temperature, sexual signals and mate choice in a desert-dwelling jumping spider. Anim. Behav. **170**, 81-87. (10.1016/j.anbehav.2020.10.010)

[14] Walker TJ. 1957 Specificity in the response of female tree crickets (Orthoptera, Gryllidae, Oecanthinae) to calling songs of the males. Ann. Entomol. Soc. Am. **50**, 626-636. (10.1093/aesa/50.6.626)

[15] Walker TJ. 1975 Effects of temperature on rates in poikilotherm nervous systems: evidence from the calling songs of meadow katydids (Orthoptera: Tettigoniidae: Orchelimum) and reanalysis of published data. J. Comp. Physiol. **101**, 57-69. (10.1007/BF00660119)

[16] Walker TJ. 1975 Effects of temperature, humidity, and age on stridulatory rates in *Atlanticus* spp. (Orthoptera: Tettigoniidae: Decticinae). Ann. Entomol. Soc. Am. **68**, 607-611. (10.1093/aesa/68.3.607)

[17] Gerhardt HC. 1978 Temperature coupling in the vocal communication system of the gray tree frog, *Hyla versicolor*. Science **199**, 992-994. (10.1126/science.199.4332.992) 17752373

[18] Gayou DC. 1984 Effects of temperature on the mating call of *Hyla versicolor*. Copeia **1984**, 733-738. (10.2307/1445157)

[19] Doherty JA. 1985 Temperature coupling and ‘trade-off’ phenomena in the acoustic communication system of the cricket, *Gryllus bimaculatus* De Geer (Gryllidae). J. Exp. Biol. **114**, 17-35. (10.1242/jeb.114.1.17)

[20] Pires A, Hoy RR. 1992 Temperature coupling in cricket acoustic communication. 1. Field and laboratory studies of temperature effects on calling song production and recognition in *Gryllus firmus*. J. Comp. Physiol. Sens. Neural Behav. Physiol. **171**, 69-78. (10.1007/bf00195962) 1403992

[21] Martin SD, Gray DA, Cade WH. 2000 Fine-scale temperature effects on cricket calling song. Can. J. Zool. **78**, 706-712. (10.1139/cjz-78-5-706)

[22] Fonseca PJ, Revez MA. 2002 Temperature dependence of cicada songs (Homoptera, Cicadoidea). J. Comp. Physiol. Neuroethol. Sens. Neural Behav. Physiol. **187**, 971-976. (10.1007/s00359-001-0267-5) 11913815

[23] Narins PM, Meenderink SWF. 2014 Climate change and frog calls: long-term correlations along a tropical altitudinal gradient. Proc. R. Soc. B **281**, 20140401. (10.1098/rspb.2014.0401) PMC3996621 24718765

[24] Symes LB, Rodríguez RL, Höbel G. 2017 Beyond temperature coupling: effects of temperature on ectotherm signaling and mate choice and the implications for communication in multispecies assemblages. Ecol. Evol. **7**, 5992-6002. (10.1002/ece3.3059) 28811890 PMC5552914

[25] Jocson DMI, Smeester M, Leith NT, Macchiano A, Fowler-Finn KD. 2019 Temperature coupling of mate attraction signals and female mate preferences in four populations of *Enchenopa* treehopper (Hemiptera: Membracidae). J. Evol. Biol. **32**, 1046-1056. (10.1111/jeb.13506) 31278803

[26] Bennet-Clark HC. 1999 Resonators in insect sound production: how insects produce loud pure-tone songs. J. Exp. Biol. **202**, 3347-3357. 10562517 10.1242/jeb.202.23.3347

[27] Montealegre-Z F, Jonsson T, Robert D. 2011 Sound radiation and wing mechanics in stridulating field crickets (Orthoptera: Gryllidae). J. Exp. Biol. **214**, 2105-2117. (10.1242/jeb.056283) 21613528

[28] Pires A, Hoy RR. 1992 Temperature coupling in cricket acoustic communication. 2. Localization of temperature effects on song production and recognition networks in *Gryllus firmus*. J. Comp. Physiol. Sens. Neural Behav. Physiol. **171**, 79-92. (10.1007/bf00195963) 1403993

[29] Ritchie MG, Saarikettu M, Livingstone S, Hoikkala A. 2001 Characterization of female preference functions for *Drosophila montana* courtship song and a test of the temperature coupling hypothesis. Evolution **55**, 721-727. (10.1554/0014-3820(2001)055[0721:cofpff]2.0.co;2) 11392390

[30] Dolbear AE. 1897 The cricket as a thermometer. Am. Nat. **31**, 970-971. (10.1086/276739)

[31] Xu MZ, Shaw KL. 2019 The genetics of mating song evolution underlying rapid speciation: linking quantitative variation to candidate genes for behavioral isolation. Genetics **211**, 1089-1104. (10.1534/genetics.118.301706) 30647070 PMC6404256

[32] Hoy RR, Hahn J, Paul RC. 1977 Hybrid cricket auditory behavior: evidence for genetic coupling in animal communication. Science **195**, 82-84. (10.1126/science.831260) 831260

[33] Cade W. 1975 Acoustically orienting parasitoids: fly phonotaxis to cricket song. Science **190**, 1312-1313. (10.1126/science.190.4221.1312)

[34] Mason AC, Oshinsky ML, Hoy RR. 2001 Hyperacute directional hearing in a microscale auditory system. Nature **410**, 686-690. (10.1038/35070564) 11287954

[35] Lee N, Mason AC. 2017 How spatial release from masking may fail to function in a highly directional auditory system. eLife **6**, e20731. (10.7554/eLife.20731) 28425912 PMC5443663

[36] Lee N, Kirtley A, Pressman I, Jirik K, Koucoulas D, Mason A. 2019 Developing a phonotaxis performance index to uncover signal selectivity in walking phonotaxis. Front. Ecol. Evol. **7**, 334. (10.3389/fevo.2019.00334)

[37] Walker TJ. 1993 Phonotaxis in female *Ormia ochracea* (Diptera, Tachinidae), a parasitoid of field crickets. J. Insect Behav. **6**, 389-410. (10.1007/bf01048119)

[38] Gray DA, Banuelos C, Walker SE, Cade WH, Zuk M. 2007 Behavioural specialization among populations of the acoustically orienting parasitoid fly *Ormia ochracea* utilizing different cricket species as hosts. Anim. Behav. **73**, 99-104. (10.1016/j.anbehav.2006.07.005)

[39] Walker TJ. 1962 Factors responsible for intraspecific variation in the calling songs of crickets. Evolution **16**, 407-428. (10.1111/j.1558-5646.1962.tb03234.x)

[40] Müller P, Robert D. 2001 A shot in the dark: the silent quest of a free-flying phonotactic fly. J. Exp. Biol. **204**, 1039-1052. (10.1242/jeb.204.6.1039) 11222123

[41] Mason AC, Lee N, Oshinsky ML. 2005 The start of phonotactic walking in the fly *Ormia ochracea*: a kinematic study. J. Exp. Biol. **208**, 4699-4708. (10.1242/jeb.01926) 16326951

[42] Lee N, Elias DO, Mason AC. 2009 A precedence effect resolves phantom sound source illusions in the parasitoid fly *Ormia ochracea*. Proc. Natl Acad. Sci. USA **106**, 6357-6362. (10.1073/pnas.0809886106) 19332794 PMC2669327

[43] Adamo SA, Robert D, Perez J, Hoy RR. 1995 The response of an insect parasitoid, *Ormia ochracea* (Tachinidae), to the uncertainty of larval success during infestation. Behav. Ecol. Sociobiol. **36**, 111-118. (10.1007/bf00170716)

[44] Adamo SA, Robert D, Hoy RR. 1995 Effects of a tachinid parasitoid, *Ormia ochracea*, on the behavior and reproduction of its male and female field cricket hosts (*Gryllus* spp.). J. Insect Physiol. **41**, 269-277. (10.1016/0022-1910(94)00095-x)

[45] Vincent CM, Bertram SM. 2010 Collection and laboratory culture of *Ormia ochracea* (Diptera: Tachinidae). J. Entomol. Sci. **45**, 1-7. (10.18474/0749-8004-45.1.1)

[46] Lott GK, Rosen MJ, Hoy RR. 2007 An inexpensive sub-millisecond system for walking measurements of small animals based on optical computer mouse technology. J. Neurosci. Methods **161**, 55-61. (10.1016/j.jneumeth.2006.10.007) 17123627

[47] Kilmer JT, Fowler-Finn KD, Gray DA, Höbel G, Rebar D, Reichert MS, Rodríguez RL. 2017 Describing mate preference functions and other function-valued traits. J. Evol. Biol. **30**, 1658-1673. (10.1111/jeb.13122) 28556474

[48] Gray DA, Kunerth HD, Zuk M, Cade WH, Balenger SL. 2019 Molecular biogeography and host relations of a parasitoid fly. Ecol. Evol. **9**, 11 476-11 493. (10.1002/ece3.5649) PMC6802024 31641487

[49] Sakaguchi KM, Gray DA. 2011 Host song selection by an acoustically orienting parasitoid fly exploiting a multispecies assemblage of cricket hosts. Anim. Behav. **81**, 851-858. (10.1016/j.anbehav.2011.01.024)

[50] Broder ED, Gallagher JH, Wikle AW, Welsh GT, Zonana DM, Firneno Jr TJ, Tinghitella RM. 2023 A well-studied parasitoid fly of field crickets uses multiple alternative hosts in its introduced range. Evol. Ecol. **37**, 477-492. (10.1007/s10682-022-10225-1)

[51] Hennig RM. 2003 Acoustic feature extraction by cross-correlation in crickets? J. Comp. Physiol. Neuroethol. Sens. Neural Behav. Physiol. **189**, 589-598. (10.1007/s00359-003-0438-7) 12879353

[52] Greenfield MD, Medlock C. 2007 Temperature coupling as an emergent property: parallel thermal effects on male song and female response do not contribute to species recognition in an acoustic moth. Evolution **61**, 1590-1599. (10.1111/j.1558-5646.2007.00140.x) 17598742

[53] Skovmand O, Boel Pedersen S. 1983 Song recognition and song pattern in a shorthorned grasshopper. J. Comp. Physiol. **153**, 393-401. (10.1007/BF00612593)

[54] Bauer M, Helversen O. 1987 Separate localization of sound recognizing and sound producing neural mechanisms in a grasshopper. J. Comp. Physiol. A Neuroethol. Sens. Neural. Behav. Physiol. **161**, 95-101. (10.1007/bf00609458)

[55] Bernal XE, Page RA. 2023 Tactics of evasion: strategies used by signallers to deter eavesdropping enemies from exploiting communication systems. Biol. Rev. **98**, 222-242. (10.1111/brv.12904) 36176190

[56] Bernal XE, Page RA. 2022 Editorial. How enemies shape communication systems: sensory strategies of prey to avoid eavesdropping predators and parasites. Front. Ecol. Evol. **10**, 989763. (10.3389/fevo.2022.989763)

[57] Lakes-Harlan R, Lehmann GC. 2015 Parasitoid flies exploiting acoustic communication of insects—comparative aspects of independent functional adaptations. J. Comp. Physiol. A **201**, 123-132. (10.1007/s00359-014-0958-3) 25369901

[58] Jirik KJ, Dominguez JA, Abdulkarim I, Glaaser J, Stoian ES, Almanza LJ, Lee N. 2023 Data from: Parasitoid-host eavesdropping reveals temperature coupling of preferences to communication signals without genetic coupling. Dryad Digital Repository. (10.5061/dryad.jwstqjqfs) PMC10427829 37583323

[59] Jirik KJ, Dominguez JA, Abdulkarim I, Glaaser J, Stoian ES, Almanza LJ, Lee N. 2023 Parasitoid–host eavesdropping reveals temperature coupling of preferences to communication signals without genetic coupling. Figshare. (10.6084/m9.figshare.c.6771479) PMC10427829 37583323

